# Bilateral Achilles tendinopathy of unknown origin responsive to corticosteroids: Case report

**DOI:** 10.1097/MD.0000000000045436

**Published:** 2025-10-24

**Authors:** Jinqi Huang, Qihong Chen

**Affiliations:** a Department of Interventional Vascular Surgery, The First Hospital of Putian City, Putian, Fujian Province, P.R. China.

**Keywords:** Achilles tendinopathy, acute, bilateral, glucocorticoid-responsive, idiopathic

## Abstract

**Rationale:**

This case presents a rare instance of bilateral acute Achilles tendinopathy with an unidentified etiology, unreported in previous literature. Documented cases of bilateral Achilles tendon pathologies have typically been associated with specific medications, chronic enthesitis, or slowly progressing xanthomas.

**Patient concerns:**

A 55-year-old male presented with fever and bilateral Achilles tendon swelling and pain. Laboratory tests showed significantly elevated inflammatory markers (C-reactive protein 96.6 mg/L, erythrocyte sedimentation rate 69.0 mm/h) and elevated immunoglobulin A (4.44 g/L). Imaging revealed fusiform swelling with inflammatory changes in both Achilles tendons. The patient had a medical history of radical prostatectomy, chronic hepatitis B virus carrier status, and familial hypercholesterolemia.

**Diagnoses:**

Bilateral acute Achilles tendinopathy of unknown etiology. Its sensitivity to corticosteroid therapy, coupled with elevated serum immunoglobulin A, suggests a possible immune-mediated mechanism.

**Interventions:**

The core treatment was systemic corticosteroid therapy (methylprednisolone, dexamethasone). Empirical antibiotics were initially administered but were subsequently discontinued due to lack of evidence for infection and the marked efficacy of corticosteroid therapy. Concurrent medications included atorvastatin for hypercholesterolemia and entecavir for hepatitis B.

**Outcomes:**

Symptoms resolved rapidly, with no recurrence during the 6-month follow-up period.

**Lessons:**

This case suggests a possible pathogenic role of immunoglobulin A-related immune mechanisms, though the lack of histopathological biopsy and genetic testing limited further etiological investigation. It offers a diagnostic and therapeutic reference for this rare bilateral acute Achilles tendinopathy, highlighting the potential role of immune factors in atypical tendinopathies.

## 1. Introduction

In our clinical practice, we encountered a rare case of bilateral acute Achilles tendinopathy with an unknown etiology, which has not been reported in similar cases in previous literature. The patient presented with fever and bilateral Achilles tendon swelling/pain as the primary clinical manifestations, and symptoms were alleviated following glucocorticoid therapy. In contrast to this case, previously reported instances of bilateral Achilles tendon pathology have often been associated with specific medications, manifested as chronic enthesitis, or presented as slowly growing xanthomas. Herein, we discuss the possible etiology and treatment strategies for this case, aiming to provide clinical references for the diagnosis and treatment of similar cases in the future.

## 2. Medical records

On September 15, 2024, a 55-year-old male patient was admitted to the hospital, with the chief complaints of “recurrent fever, dizziness, and bilateral Achilles tendon swelling and pain for half a month.” His medical history included a radical prostatectomy 15 months prior, with no adjuvant therapy administered postoperatively and regular follow-ups showing no abnormalities. He experienced intermittent mild swelling in both lower legs postoperatively. He was also a chronic carrier of hepatitis B virus, receiving regular entecavir therapy for 4 years, and had familial hypercholesterolemia with total cholesterol levels maintained at 6 to 8 mmol/L. He had previously been treated with statins but discontinued them in recent years. Physical examination on admission revealed afebrile but with ambulatory difficulty, symmetrical erythema and swelling in the bilateral Achilles tendon regions with marked tenderness, and mild erythema/swelling in the distal lower legs. Laboratory tests showed a white blood cell count of 10.07 × 10^9^/L (normal range: 3.5–9.5 × 10^9^/L), neutrophil percentage of 78.2% (normal range: 40%–75%), erythrocyte sedimentation rate (ESR) of 69.0 mm/h (normal range: 0–15 mm/h), C-reactive protein (CRP) of 96.6 mg/L (normal range: 0–8 mg/L), total cholesterol of 7.82 mmol/L (normal range: 3.30–5.20 mmol/L), procalcitonin of 0.14 ng/mL (0–0.5 ng/mL), and negative blood culture. Total prostate-specific antigen was 0.031 ng/mL (0–4 ng/mL). A chest computed tomography scan revealed no significant abnormalities. The patient was treated with methylprednisolone sodium succinate, cefoperazone–sulbactam, atorvastatin calcium, and entecavir.

On September 16, symptoms improved, allowing normal walking, although redness and swelling persisted in the bilateral Achilles tendon regions. Color Doppler ultrasound indicated significant thickening of the bilateral Achilles tendons with heterogeneous echo and hypoechoic areas within, along with abundant blood flow signals, indicative of tendinopathy.

On September 17, methylprednisolone sodium succinate was discontinued, and cefoperazone–sulbactam was discontinued on September 18.

On September 21, symptoms recurred, with difficulty in walking. Human leukocyte antigen B27 (HLA-B27), antinuclear antibodies, extractable nuclear antigen antibodies, anti-double-stranded DNA antibodies, anti-cyclic citrullinated peptide antibodies, and complement C3/C4 were all negative. A pelvic computed tomography scan with contrast and a plain pelvic X-ray revealed no significant abnormalities. Dexamethasone sodium phosphate and cefoperazone–sulbactam were administered.

**Figure 1.**
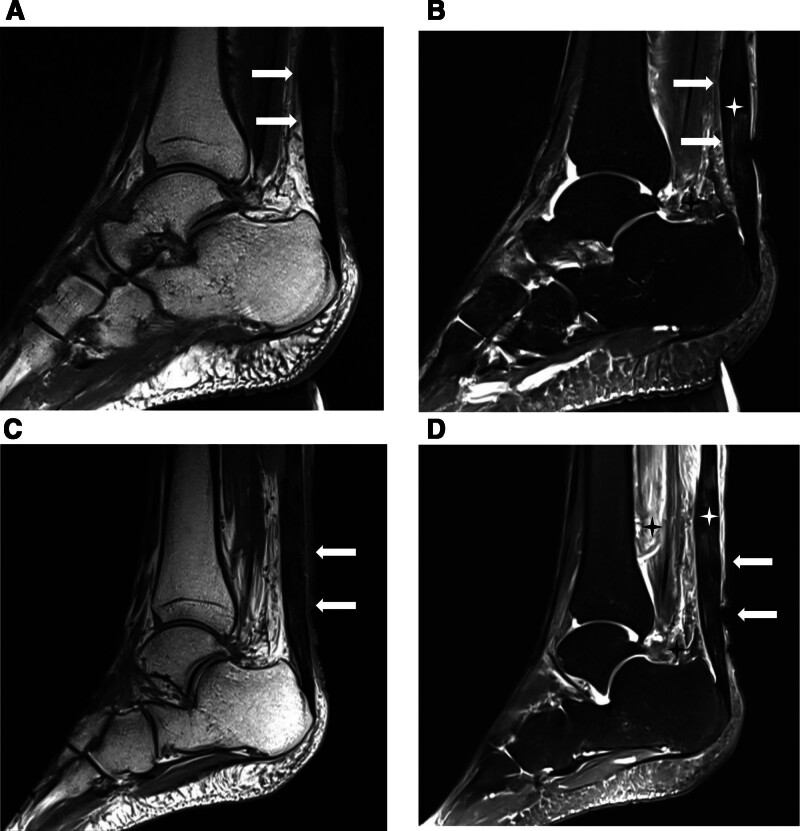
MRI findings of bilateral ankle joints. The bilateral Achilles tendons demonstrate symmetrical fusiform swelling (arrows) with signal abnormalities (white asterisks). Diffuse abnormal signal shadows (black asterisks) are observed in the soft tissues of the distal lower legs and those surrounding the ankle joints, consistent with acute Achilles tendinopathy accompanied by peritendinous inflammatory changes (A: left T1-weighted imaging; B: left T2-weighted fat-suppressed imaging; C: right T1-weighted imaging; D: right T2-weighted fat-suppressed imaging). MRI = magnetic resonance imaging.

On September 22, symptoms improved, allowing normal walking. An magnetic resonance imaging scan indicated fusiform swelling of the bilateral Achilles tendons with signal abnormalities and diffuse abnormal signal shadows in the soft tissues surrounding the lower segments of both lower legs and ankles, suggesting inflammatory lesions (Fig. 1A–D). An echocardiogram revealed no significant abnormalities.

On September 23, immunoglobulin (Ig)A was 4.44 g/L (normal range: 0.69–3.8 g/L), ESR was 94.0 mm/h, CRP was 20.10 mg/L, white blood cell count was 7.36 × 10^9^/L, neutrophil percentage was 73.5%, IgM was 0.41 g/L (0.63–2.77 g/L), and IgG was 14.50 g/L (7.24–16.85 g/L). Tests for antistreptolysin O, rheumatoid factor, human immunodeficiency virus antigen–antibody, syphilis spirochete antibody, tuberculosis T-cell detection, brucellosis antibody, carbohydrate antigen (CA) 199, CA 125, CA 153, carcinoembryonic antigen, alpha-fetoprotein, abnormal prothrombin, and bacterial culture were all negative. Cefoperazone–sulbactam were discontinued.

On September 30, symptoms resolved, and the patient was discharged, with ESR at 38.0 mm/h and CRP at 0.39 mg/L. Post-discharge, the patient was treated with methylprednisolone tablets, gradually tapered and discontinued after 1 month, and continued long-term treatment with atorvastatin calcium and entecavir.

## 3. Discussion

The patient presented with intermittent bilateral calf swelling following radical prostatectomy, suggesting impaired lymphatic drainage secondary to pelvic lymph node dissection. The current episode was characterized by recurrent fever, bilateral Achilles tendon pain and swelling, and erythema in the lower segments of both calves, accompanied by elevated inflammatory markers, which raised suspicion of bacterial infection. However, negative blood cultures, normal procalcitonin levels, bilateral symmetric involvement of the Achilles tendons, and a favorable response to corticosteroid therapy ruled out a diagnosis of bacterial infection. Negative results for T-cell-based tuberculosis infection tests and brucellosis antibodies in this case also excluded specific pathogen-induced infections. The absence of HLA-B27 positivity and radiographic changes in the sacroiliac joints or spine ruled out spondyloarthritis. Furthermore, comprehensive autoimmune antibody testing (including antinuclear antibodies, extractable nuclear antigen antibodies, anti-double-stranded DNA antibodies, and anti-cyclic citrullinated peptide antibodies) yielded negative results. This finding is crucial for differential diagnosis, as it effectively rules out common rheumatic diseases typically characterized by these serological markers, such as systemic lupus erythematosus, Sjögren syndrome, systemic sclerosis, and rheumatoid arthritis.

Although the patient had a history of familial hypercholesterolemia, the acute inflammatory presentation and diffuse bilateral Achilles tendon swelling rather than localized masses did not support a diagnosis of simple tendon xanthoma. Regular follow-up examinations after radical prostatectomy showed no abnormalities, thus excluding the possibility of a paraneoplastic syndrome associated with prostate cancer. The absence of skin or nail lesions in this case made a diagnosis of psoriatic arthritis unlikely. The bilateral symmetric involvement of the Achilles tendons, coupled with their sensitivity to corticosteroid therapy, suggested an immune-mediated inflammatory process more consistent with an autoimmune/inflammatory disorder. In summary, the clinical presentation, steroid responsiveness, and elevated IgA are suggestive of an IgA-related immune-mediated tendinopathy, though definitive evidence is lacking in the absence of histopathological confirmation. The relationship between immune responses and diseases is widespread across various organisms.^[1]^ The pathogenesis in this case may be similar to that of IgA-mediated autoimmune diseases, such as IgA vasculitis or IgA nephropathy.

The treatment regimen for this case centered around glucocorticoids, combined with comprehensive interventions including antibiotics and lipid-lowering medications, which yielded significant clinical improvement, evidenced by the alleviation of symptoms and reduction in inflammatory markers. Glucocorticoids can rapidly suppress noninfectious inflammation. Although bacterial infection was not the primary consideration, empirical antibiotics were initially prescribed due to the patient’s systemic inflammatory response (CRP 96.6 mg/L). However, the decision to ultimately discontinue antibiotics was based on the following rationale: First, the clinical presentation was inconsistent with a typical infection. Bilateral and symmetrical involvement of the Achilles tendons is exceedingly rare in infectious diseases, as soft tissue infections in the lower limbs are predominantly unilateral. Second, there was insufficient microbiological evidence—blood cultures were negative, and the procalcitonin level was within the normal range (0.14 ng/mL). Third, and most critically, the patient’s therapeutic response strongly suggested sterile inflammation. He exhibited a highly sensitive “on-off effect” in response to glucocorticoid therapy, with symptoms rapidly resolving upon administration and recurring promptly after discontinuation. This pattern starkly contrasts with the gradual improvement over several days typically required for antibiotics to take effect. Collectively, these findings cast doubt on the necessity of antibiotic use and highlight the challenges in distinguishing between sterile inflammation and infection in acute clinical settings. The patient had familial hypercholesterolemia, and long-term statin therapy was administered to mitigate the associated risks arising from hypercholesterolemia.

Currently, clinical reports on bilateral Achilles tendinopathy are relatively scarce, with all existing literature comprising individual case reports that exhibit notable discrepancies from the present case in terms of etiology, clinical manifestations, and imaging characteristics. Previous studies have documented cases of bilateral Achilles tendinopathy and even tendon ruptures induced by fluoroquinolone antibiotics and glucocorticoid use.^[2–4]^ Olivieri et al reported a case of HLA-B27-associated bilateral Achilles tendinopathy, which presented as a chronic course and showed no response to nonsteroidal anti-inflammatory drugs.^[5]^ Rizvanoğlu et al and Bussey et al have separately reported cases of sitagliptin-induced bilateral Achilles tendinopathy, with imaging findings suggesting lesions at the insertions of the bilateral Achilles tendons.^[6,7]^ Moniri et al reported a case of statin-associated Achilles tendon rupture and bilateral tendinopathy, with magnetic resonance imaging indicating severe thickening and tendinopathy in the repaired Achilles tendon.^[8]^ Carranza-Bencano et al reported a case of bilateral Achilles tendon xanthoma as the initial manifestation of familial hypercholesterolemia, characterized by the slow growth of tumors in the bilateral Achilles tendon regions over several years, accompanied by pain and deformity.^[9]^

The etiology of bilateral acute Achilles tendinopathy in this case remains undetermined; however, its sensitivity to glucocorticoid therapy suggests the possible involvement of an immune-mediated mechanism. Although common causes have been excluded, the elevated serum IgA level may be indicative, necessitating long-term follow-up to rule out underlying immune-related disorders. A limitation of this case is the absence of tendon biopsy and relevant genetic testing, which prevented further clarification of the etiology.

## Author contributions

**Conceptualization:** Jinqi Huang, Qihong Chen.

**Data curation:** Jinqi Huang, Qihong Chen.

**Investigation:** Jinqi Huang, Qihong Chen.

**Methodology:** Jinqi Huang, Qihong Chen.

**Writing – original draft:** Jinqi Huang, Qihong Chen.

**Writing – review & editing:** Jinqi Huang, Qihong Chen.
